# Short-interval fires increasing in the Alaskan boreal forest as fire self-regulation decays across forest types

**DOI:** 10.1038/s41598-022-08912-8

**Published:** 2022-03-22

**Authors:** B. Buma, K. Hayes, S. Weiss, M. Lucash

**Affiliations:** 1 Department of Integrative Biology, University of Colorado, Denver, CO USA; 2 University of Oregon, Eugene, OR USA

**Keywords:** Boreal ecology, Climate-change ecology, Fire ecology, Forestry, Natural hazards

## Abstract

Climate drivers are increasingly creating conditions conducive to higher frequency fires. In the coniferous boreal forest, the world’s largest terrestrial biome, fires are historically common but relatively infrequent. Post-fire, regenerating forests are generally resistant to burning (strong fire self-regulation), favoring millennial coniferous resilience. However, short intervals between fires are associated with rapid, threshold-like losses of resilience and changes to broadleaf or shrub communities, impacting carbon content, habitat, and other ecosystem services. Fires burning the same location 2 + times comprise approximately 4% of all Alaskan boreal fire events since 1984, and the fraction of short-interval events (< 20 years between fires) is increasing with time. While there is strong resistance to burning for the first decade after a fire, from 10 to 20 years post-fire resistance appears to decline. Reburning is biased towards coniferous forests and in areas with seasonally variable precipitation, and that proportion appears to be increasing with time, suggesting continued forest shifts as changing climatic drivers overwhelm the resistance of early postfire landscapes to reburning. As area burned in large fire years of ~ 15 years ago begin to mature, there is potential for more widespread shifts, which should be evaluated closely to understand finer grained patterns within this regional trend.

## Introduction

Fire dominates landscape dynamics and change in the world’s largest terrestrial biome^1^, the boreal forest. Fire disturbance has been a functioning part of the boreal ecosystem for at least 10,000 years^2^, and many of the species in this ecosystem are evolved for and dependent on relatively regular but relatively infrequent burning (e.g., 75–100 + years between events). Variations in fire frequencies can drive local landscape changes in species composition, landcover and wildlife habitat. The boreal forest system also stores an immense amount of carbon (367–1716 Pg.^3^). Fire can threaten that carbon reservoir, which has built up over millennia^4^. Increasing fire frequency combusts and erodes the old carbon of boreal forest soils^5^, resulting in a potential net flux into the atmosphere. Therefore, changes in fire frequency and severity as a result of climate change are an important interest to global climate change^6^.

Multi-year trends in boreal fire activity can be divided into two broad categories of control: top-down forcing driven by climate and bottom-up driven by vegetation (fuel) composition and quantity^7^. These two drivers come together (with climate felt through the weather at time of event) to influence fire behavior. Climate forcing on fire frequency in the boreal is increasing rapidly and generally in a positive direction; longer snow-free seasons^8^, warmer temperatures, and higher moisture stress are all conducive to increased burned area^9^. Together, changes in these drivers may increase fire prevalence in the boreal forest. The bottom-up controls on fire frequency, however, are less clear in terms of their magnitude and direction, because they differ based on forest type. Coniferous forests are generally highly flammable but with specialized resilience mechanisms (e.g., black spruce, *Picea mariana*, with serotinous cones) and a thick organic layer that facilitates coniferous regeneration; as a result, they are well suited to the historical fire regime^10^. Broadleaf trees are less flammable due to higher fuel moistures, differing structure, and establish proportionally better on mineral soil (e.g., *Betula spp* and *Populus spp*^11^). The dynamic interplay between climate, fuel type, and fire has created a shifting mosaic of vegetation ages and types over the last 10,000 years, but generally favored coniferous vegetation^2^.

### Short-interval fires and cumulative severity on ecosystems

Although the relative proportion of coniferous to broadleaf cover can also vary as a function of localized topographic, climatic, and edaphic factors, of prime concern here is the feedback between increasing fire frequency and potentially long-term, fundamental regime shifts from coniferous to broadleaf forests (which we define as a loss of conifers, not simply changes in relative dominance post-fire). The concept of reburning and compound disturbances as “unique” entities, more than the sum of their individual-fire parts, has emerged over the past decade^12–14^. Short intervals between fires could cause rapid shifts from coniferous forests to broadleaf forests over large extents if significant burns (e.g., near 100% mortality) occur with an interval less than the time required to rebuild the serotinous aerial seedbank^10,15–17^.

Briefly, if a second fire occurs prior to the reconstruction of serotinous cones, there is little seed supply available for regeneration^16,18^ and dispersal from unburned edges is generally less likely due to the relatively heavy seeds of conifers^16^. There is ample empirical evidence for this; short-interval fires with intervals less than 30-year intervals (less than the typical maturity time of black spruce of 30–40 years^19^ have repeatedly resulted in a near or complete loss of black spruce seed and seedlings (e.g.,^15,20^). Other serotinous species show similar population declines after short-interval fires as well ^13,14,18^. This creates a situation uniquely different from high-severity single burns, which can also result in shifts to broadleaf *dominated* stands, at least initially. However, those situations still generally have ample coniferous regeneration underneath the faster growing broadleaf species^21^. Thus they retain the potential for long-term coniferous forest system recovery (e.g., based on succession studies^22^ or as inferred from the paleorecord^2^), though note that this is relatively unstudied and their long-term fate in future climates unknown. If conifers are absent, however, a coniferous forest recovery is less likely.

Repeat burns have a cumulative severity aspect as well. Short-interval fires reduce remnant organic material, soil, and litter layers, continually reducing organic soil with each burning^20,23,24^. Broadleaf seeds, which are better dispersers and strong competitors on mineral seedbeds, can more easily invade and dominate^21,25^. As a result of both seed limitations and cumulative severity impacts, areas which have been relatively stable as coniferous forests with 100–200 year fire return intervals can shift to broadleaf dominance with little to no coniferous presence with only a single short-interval event (e.g., over 10 years).

### Negative feedbacks

Although the occasional fire has burned through recovering post-fire stands, this is generally thought to be uncommon throughout the Alaskan boreal forest. There are several intrinsic constraints on flammability that limit reburns (essentially, bottom-up controls on fire occurrence). Young, less flammable postfire stands can act as a negative feedback against increased burn rates despite a warming and drying climate, even at broad scales (e.g., boreal Canada^26^; western continental US^27^). The mechanisms behind this feedback are generally a function of less available fuels and less flammable fuel types (broadleaved shrubs). Although postfire stands have significant downed debris that can function as fuel, canopy fuel loads are typically low compared to mature conifer stands^28^. Despite this, extreme conditions can drive fire through recently burned locations, consuming dead wood, detritus, and recovering vegetation, and in major fire years with extreme weather, vegetation type does not appear to strongly limit burn patterns within a fire^29^.

### Unknowns

Despite the well-documented potential for rapid regime shifts from field-based studies, little is known about the regional scale patterns in short-interval fires in the boreal. It is still relatively unknown how common short-interval fires are, if they are increasing, and where they are increasing. Evidence from the Canadian boreal^26^ and other forested ecosystems^27^ suggest that, at least for the time period of available data, negative feedbacks are likely still significant. Comparisons with paleo records also suggest a rough similarity between rates of short-interval fires now and in the lake sediment charcoal records, (e.g.,^2^), though the relatively coarse resolution in sediment cores (~ 50 years) constrains confidence. Finally, it is unclear if short-interval fires are biased towards flammable vegetation types or not, which would influence the potential for broad-scale type conversions. In general, we would predict increasing rates of reburning simply due to chance; as fire frequency increases, reburning should increase as well. However, the relative strengths of the top-down, intensifying climatic/weather drivers of fire in the boreal vs the negative fuel-driven feedbacks are unknown. The significance of the global boreal forest ecosystem to climate, habitat, human use, and other factors makes understanding the potential for regime shifts a research imperative.

### Questions and predictions

The following questions were asked, (1) Is the proportion of short-interval burns increasing, (2) is there evidence for negative feedbacks in reburn occurrence, and over what timescales, (3) in what vegetation types are those occurring and is the proportion of coniferous forest burned in short-interval events changing, and (4) are short-interval fires occurring in predictable climate or topographic locations in the region, or are they distributed randomly?

## Results

### How common are short-interval fires?

**Figure 1:**
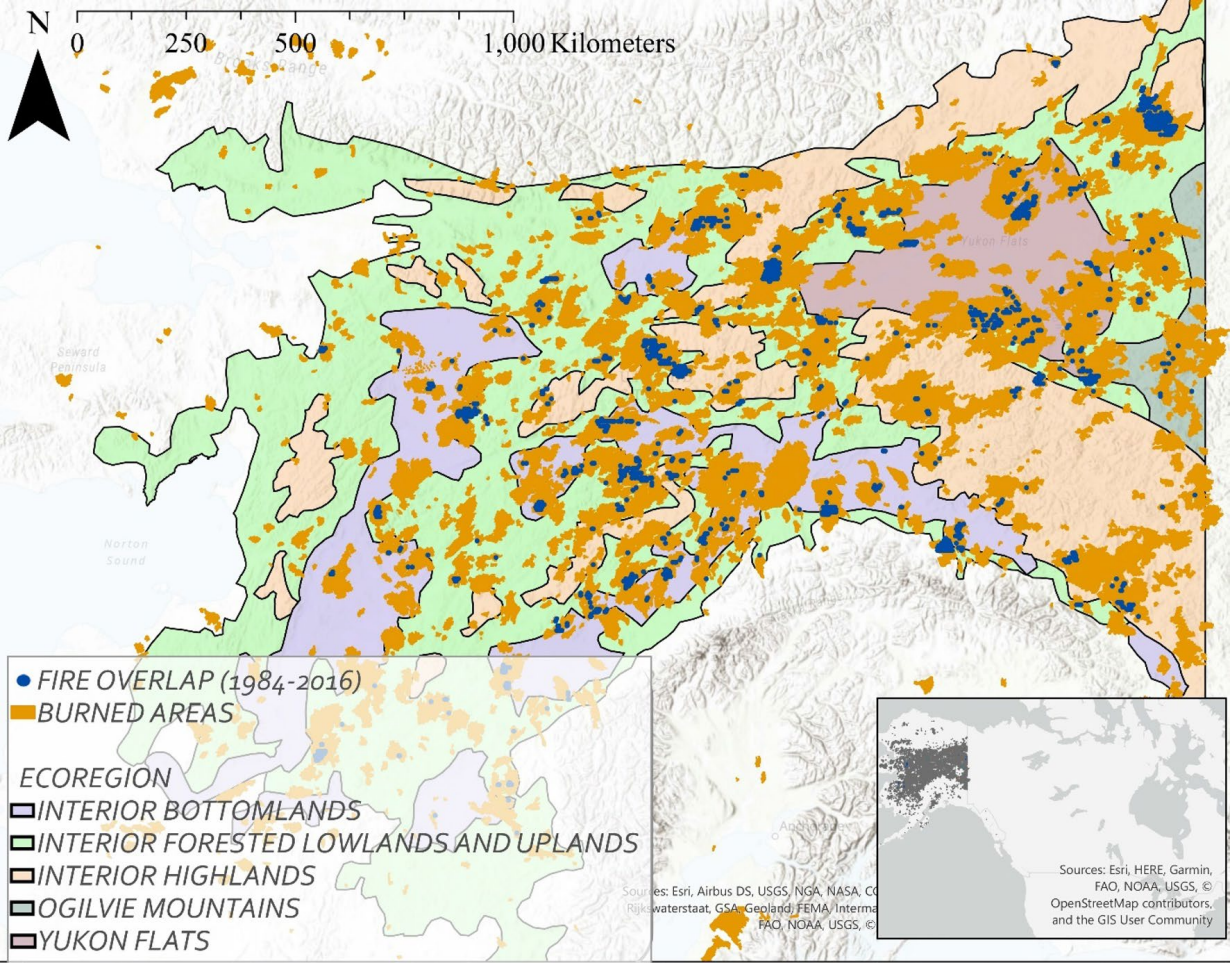
The study area spans the entirety of interior Alaska. Fires burned in the satellite record (Landsat and MTBS program), locations of overlapping fires (short-interval fires, 2 +) and ecoregions denoted. Inset shows fire footprints within North America. Map created in ESRI ArcPro 2.5.1.

**Figure 2:**
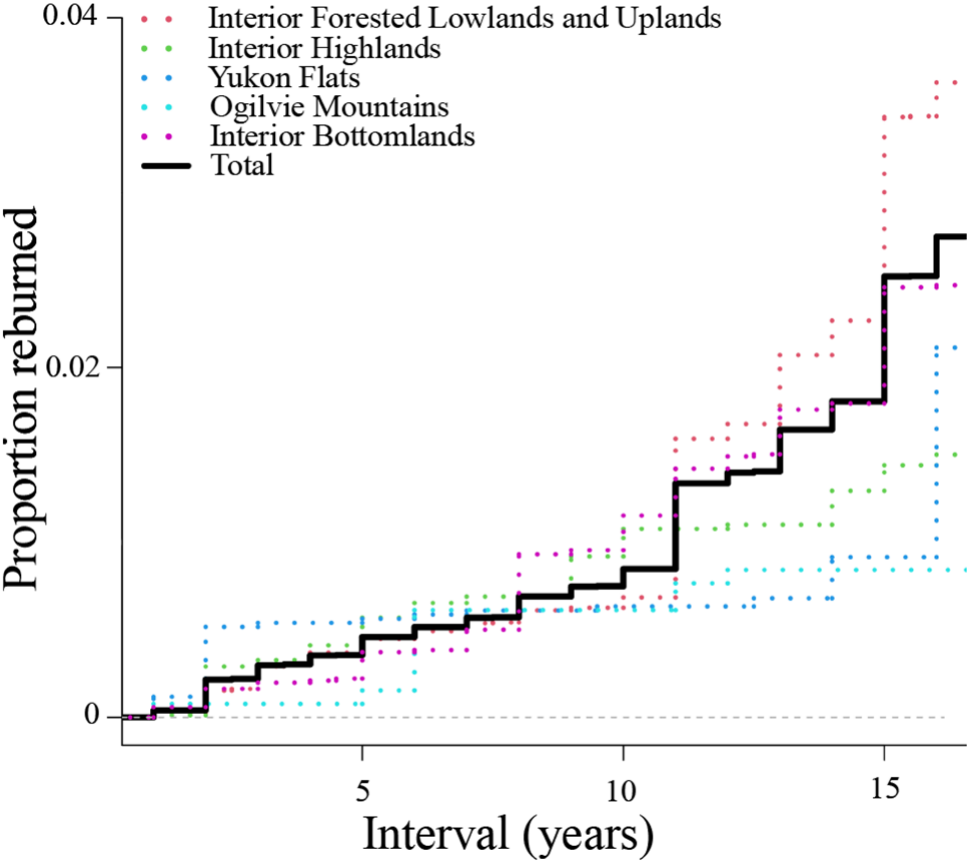
Empirical cumulative distribution functions for short-interval fires as a function of years, 1984 to 2016 inclusive. Few fires are observed with intervals ≤ 10 years, at which point they become much more common. Intervals > 16 years not shown as there is increasingly less opportunity for those to occur in the dataset due to lack of data, skewing the potential proportions (e.g., it is increasingly downwardly biased). With that caveat, the entire dataset can be seen in Supplementary Fig. S1.

Approximately 20% of the Alaskan landscape burned in the 32 years of observation (Fig. 1). The majority of fires were single burns; locations that experienced 2 + fires (reburns over any interval) in that observational period made up ~ 4% of all fires (Table 1). In general, there were very few reburns within the first 10 years after a fire, at which point the likelihood of reburning increases fairly rapidly, especially in the interior forested ecoregion (Fig. 2). 90.5% of reburns occur ≥ 5 years after the initial fire, 80.0% of reburns occur ≥ 10 years after the initial fire.

**Table 1:** Observed rates of burning and reburning within the entire satellite remote sensing period. Burn percentage overall refers to the ecoregion or overall landscape as a whole. Single, 2, and 3 percentages refer to burned fractions only.

Ecoregion	Burn % (overall)	Single fire %	2 fires %	3 fires %
Interior forested lowlands/uplands	18.7	95.5	4.3	0.1
Yukon Flats	29.5	95.5	4.4	0.1
Interior bottomlands	21.3	96.4	3.6	0.05
Interior highlands	17.3	97.7	2.2	0.04
Ogilvie Mountains	29.2	99.0	1.0	0
Overall	19.8	96.2	3.7	0.1

### Are reburns increasing and are there signs of negative feedbacks?

**Figure 3:**
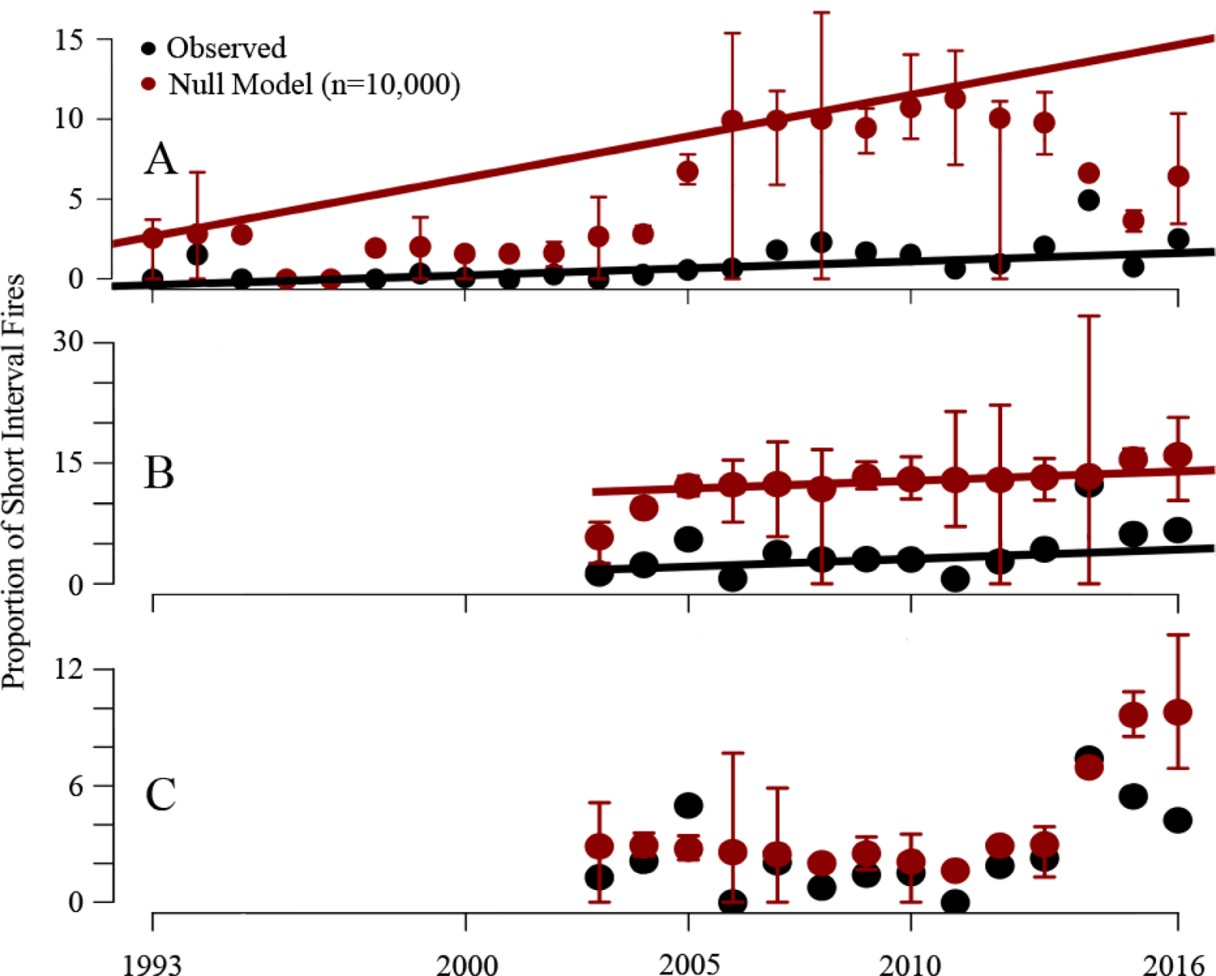
Proportion of fires observed in a given short interval and expected proportion in the absence of any spatial interaction/negative feedback (null model). (**A**) 10 year or less interval, showing general lack of increase in observed short-interval events despite increasing fires. (**B**) 20 year or less interval, where reburns are increasing at a similar rate to overall fires (suggesting a lack of negative feedbacks), but are lower, likely because the 0–20 year span integrates the < 10-year feedbacks in (**A**). (**C**) Isolating only 10–20 year intervals to show congruence between data points. Bars shown are the 25th/75th quantiles from the null model.

In general, the proportion of fires that are reburns is becoming more common as fires increase over time. However, extremely short intervals (< 10 years) are much less frequent and increasing much more slowly, and in only a slightly positive direction (0.09% per year, SE = 0.03, 5th/95th CI: 0.04–0.14) than would be expected by chance (0.52% per year, SE = 0.08, 5th/95th CI: 0.38–0.66). The mean and 25th/75th quantiles associated with the null model were also generally well above the observed proportions of reburns, only rarely overlapping with the observed rates (Fig. 3A). Exceptionally variable fire years, like 2008, 2012, and 2014, were the exceptions. In contrast, for reburns with < 20 years between fires (Fig. 3B), the median rate of increase is about twice as fast (though more variable as a result of the smaller sample size: 0.19% per year, SE = 0.20, 5th/95th CI: −0.16–0.54) and essentially the same as the rate of increase expected from the null model (0.21%, SE = 0.13, 5th/95th CI: -0.02–0.43), though the actual proportion was consistently lower as a result of integrating across the entire period (intervals from 1 to 20 years, compare the proportions of null expectations and observed in Fig. 3C, which are generally very similar).

### How common are short-interval fires in coniferous environments?

**Figure 4:**
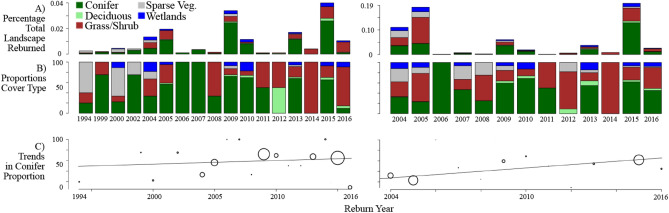
Trends in reburning by cover type for two reburning windows, ≤ 10 years (left) and ≤ 20 years (right). (**A**) Percentage of the total landscape that has burned in a reburn (2 + fires) by year of second burn. Note differing y-axis scales. (**B**) Proportion of reburns by cover type. Note that for some years no reburns in the interval were recorded, so the scale is discontinuous (≤ 10 year group). (**C**) Trend in conifer proportion burned over time (linear regression, weighted by number of observations in a year, noted with graduated circles: larger circles indicate more fires in that year). For the ≤ 10 year interval, there is no trend. For the ≤ 20 year interval, the trend is significant. Range limited to either 10 or 20 years after initial observation to decrease bias due to fires prior to the observation period.

The proportion of 2 + fire events occurring in coniferous forest areas ranged from 100% of all reburns in a given year to 0% in some low-fire years, but generally ranged around 30–85% (Fig. 4). For extremely short-interval fires < 10 years apart, there was no trend in the proportion of conifers reburned (~ 0% change, r^2^ = 0.03, F 0.43_1,15_, p = 0.5). However, when expanding to intervals of up to 20 years, the positive correlation between elapsed time and the proportion of conifers being reburned on the landscape is strong (3% increase per year; r^2^ = 0.53, F = 14.65_1,11_, *p* = 0.003). In general, this increasing proportion of coniferous short-interval fires correlates with a decrease in the proportion of sparsely vegetated landscapes (though note that coniferous forests also comprise the majority of the landscape, so some effect due to chance is also expected). Only a small number of short-interval fires were noted in originally broadleaf forests, generally < 5% of the total (and often zero), so no trend analyses were considered appropriate.

### Are short-interval fires occurring in predictable locations, and what are the characteristics of those locations?

**Figure 5:**
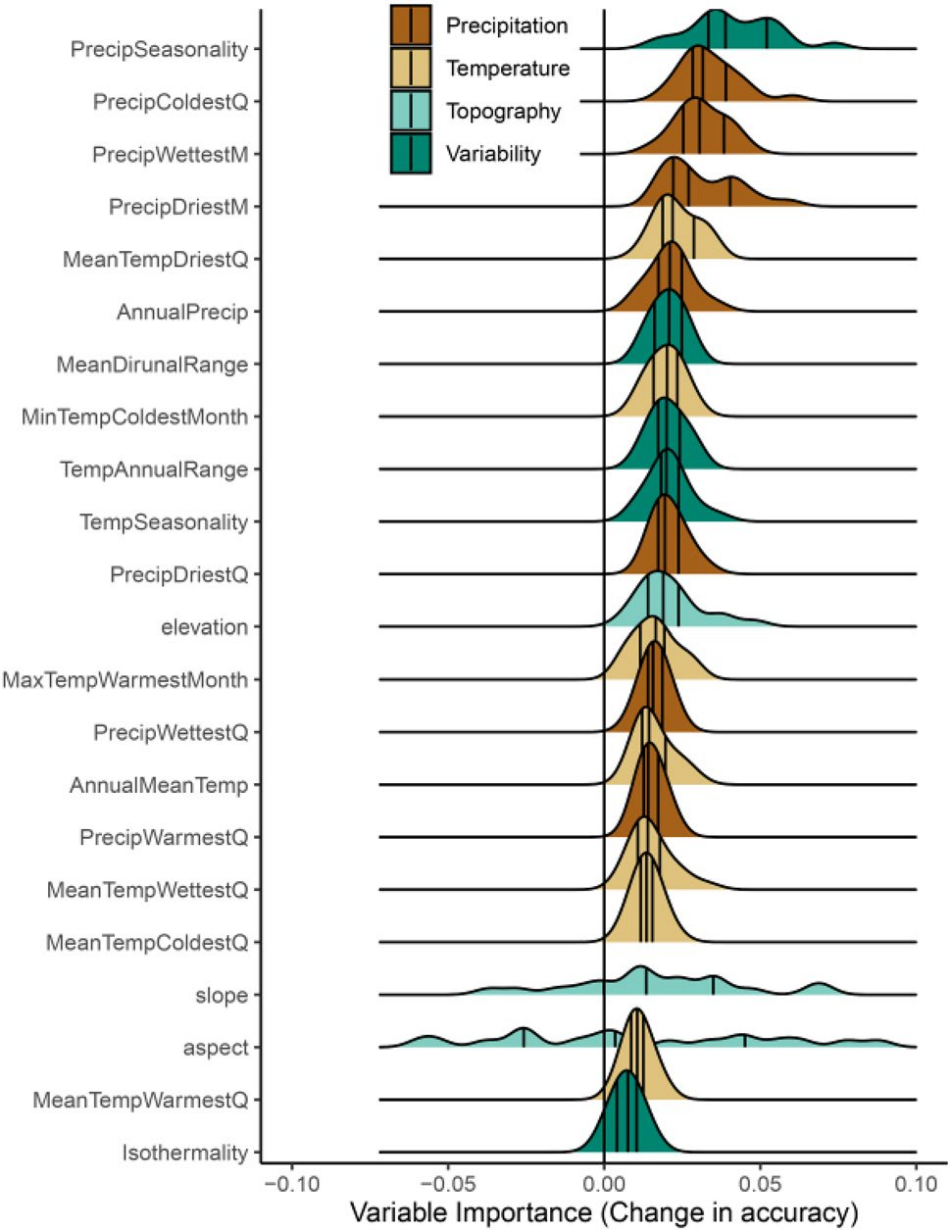
Distributions of partial variable importance scores for each climatic and topographic variable (ordered by medians). Precipitation metrics, both absolute and variability, are generally more important than temperature; topography is relatively unimportant. Colors are broad groupings of drivers for discussion. Vertical lines are 25th, median, and 75th percentiles respectively. “Q” in the variable names refers to quarter of the year, e.g., PrecipColdestQ is the precipitation of the coldest quarter of the year.

The conditional random forest model was able to distinguish between one and two fire locations with good accuracy, with a mean of 0.77 (range: 0.72–0.80), on the independent test set. In general, variable importance metrics suggest that precipitation amounts in various seasons were the most valuable parameters in distinguishing areas of short-interval fires vs. single fires in the records. Variability (except precipitation variability) was less important, and surprisingly, topographic variation was similarly non-important (Fig. 5).

## Discussion

Much of boreal interior Alaska has burned in the satellite era, and a fraction reburned. The results here indicate that short-interval fires are uncommon with intervals < 10 years and their rates seemingly insensitive to climate change to date, suggesting bottom-up fuel limitations still constrain top-down climate forcing. However, beyond that interval, the percentage of area reburning after a short interval is increasing at a similar rate to the percentage of the state burning overall, indicating that climate is associated with increasing short-interval events.

Over 4% of burned locations have burned at least twice in 32 years. This three-decade average is not dramatically different from paleo records^2^.

There appears to be strong negative feedbacks in the first decade, with few reburns observed (Fig. 2) and little change in the proportion of reburns despite increasing fire overall (Fig. 3A). Paleoecological data suggests that historically, increases in fire-climate drivers were more associated with increasing burned biomass rather than increased fire frequency^2^, further evidence that the bottom-up constraints on fire activity are fairly robust to changes in climate. However, this appears to decline after 10 years, and by 20 years post-fire, the strength of these negative feedbacks appear to be substantially reduced. The proportion of reburn events is increasing at approximately the same rate as the rate of fire overall. This can be interpreted as a signal of little self-regulation on fire behavior resulting from previous burns—the loss of bottom-up control as top-down drivers become dominant. The implications of this declining negative feedback are significant—areas recovering from burning in the large fire years of 2004 and 2005 are now increasingly vulnerable to reburning (Supplementary Fig. S1). There are signs that resistance is not completely lost early in that second decade at least—reburn frequency in 2015 and 2016 was slightly less than anticipated based on the null model for the 10–20 year interval (Fig. 3C). However, the increasing proportion of reburns in that time period suggests the feedback is still declining as the forests age, even as early as that second post-fire decade. This is long before the canopy seedbank (and thus the primary fire resilience mechanism) is recovered, explaining the observed loss of seedlings (e.g.,^20^).

There were no trends in the proportions of coniferous locations burning at intervals < 10 years, which further suggests that such short intervals are rare (Fig. 2), steady (Fig. 3A), and occurring generally at random with respect to original vegetation (Fig. 4). Although the temporal scale here precludes causal investigation, and the influences of large fire and large reburn years (one is not necessarily the same as the other) are difficult to disentangle, the lack of predictability in cover type reburning is interpreted as a function of truly extreme fire weather events that are rare in both space and time, strong enough to overwhelm the bottom-up factors constraining fire occurrence in post-fire stands.

However, when the negative feedback begins to break down after two decades, the proportion of coniferous forest burning 2 + times increases rapidly. This could come about through two, non-mutually exclusive pathways. First, the strong influence of vegetation type on reburning probabilities could constrain fire activity; recovering broadleaf forests and other ecosystem types are typically less likely to burn, and so an increase in short-interval fires driven by climate would be disproportionately felt by coniferous forests. Second, it could be that incidents of extreme conditions in top-down fire drivers, like exceptionally hot and dry conditions in some years, are biased towards areas already dominated by conifer cover. Both seem potentially likely. Although broadleaf forests can burn after short periods of time as well^20^, it appears to still be rare under the current climate (Fig. 4), though under future climates that strong feedback may yet be overwhelmed^30^. In terms of where they occur, short-interval fires are most correlated with drier and highly seasonal precipitation (large differences between driest and wettest seasons) which could favor coniferous vegetation (Fig. 5). Topography was surprisingly unimportant, despite fine-scale but significant differences in fuel moisture as a function of slope and aspect (personal observation). It seems likely that as the top-down drivers promoting fire continue to increase^6^, the window of time where negative feedbacks dominate will shrink.

The impact of conversion on future fire frequency remains relatively unknown. Paleoecological studies (e.g.,^31^) and more recent ecological studies (e.g.,^26,32^) suggest that coniferous conversion to broadleaf or open landscapes may partially alleviate the increase in top-down climate forcing. However, recent studies have hinted that more open landscapes may be more conducive to fires, likely through more rapid drying and an increase in fine fuels, though the studies are not located in the same region (Kenai region and spruce beetle triggered conversion to grasslands:^33^; tundra burning and shrub vs. grassland fires:^34^). One study which did investigate three fires in short succession in coniferous forests hints that these transitions could occur in boreal forests as well. After two fires, coniferous density did drop and broadleaf tree dominance increased (as anticipated), but after a third fire in those recovering, now-broadleaf stands, the broadleaf tree density also dropped and grass density increased^20^. Further work targeted at changes in the mechanisms underlying bottom-up resistance to fire is necessary to determine the timescales and landscape contexts in which a negative feedback might be expected to last versus those where those mechanisms will likely be overwhelmed by climate drivers in a warming world.

Although this multipronged temporal and spatial investigation into boreal forest fire dynamics is novel, there are multiple limitations to causal inference that must be kept in mind. The short time period of observation is a constraint, especially for quantifying characteristics of longer intervals in as unbiased a fashion as possible—although the satellite record gives more than three decades of fire history in a spatially repeatable fashion, the number of potential reburns for any given interval decreases as the interval observed increases and must be accounted for (true for any temporally constrained study). This can be seen in the positive best estimates for trends, but somewhat wide confidence intervals for the longer time periods (e.g., 0–20 years) Major fires in extreme conditions can also overwhelm any bottom-up controls (such as the large fire years of 2004, 2005, and 2015; see^35^ for similar phenomena with 1988 Yellowstone fires), and thus they add variability in response when aggregating general trends, as we do here. Links with paleoecological datasets could provide this important temporal perspective if higher resolution datasets could be created. We did not investigate a gradient of initial fire severity; an excellent question is the role of initial fire severity on the likelihood of reburning. Linking with similar studies in Russian boreal forests (i.e.,^36^), where species are different but many of the mechanisms similar, would be a valuable test case. Further, results from any broad scale study must continue to be linked with fine-grained, mechanistic work on the mechanisms behind regional trends.

## Conclusions

Fires are increasing globally, and their ability to drive significant shifts in ecosystem and carbon dynamics in the boreal forest via reburning (either through cumulative severity or through elimination of seed sources) makes it imperative that we understand the pattern, process, and trends in short-interval fire events. Extremely short-interval fires (< 10 years) appear to be relatively insensitive to climate change-driven increases in fire probability, but after two decades that feedback lessens. Short-interval fires at the multidecadal scale are increasing, and they are predominantly occurring in coniferous forest landscapes. This decay of fire self-regulation is important and is now occurring well within the timeframe that many would consider a short-interval fire (e.g., < 50 years). Given the ample evidence of short-interval fires causing forest conversions, these results highlight the potential for increasing rates of coniferous forest loss driven by climate change influences on fire behavior, especially under drier conditions and hotter summers. This work, which is regional in scope, needs to continue to be paired with local, mechanistic investigations to understand the causes, rates, and trends in fire resistance in burned environments.

Given that the time frame here, approximately three decades, is within the window for significant type conversions indicated by multiple field studies (e.g.,^14–16,20^), and that the proportion of coniferous forests reburning does appear to be increasing, this suggests a potential for ongoing and increasing coniferous forest loss with the associated changes in permafrost stability, carbon stocks, habitat, and other considerations.

## Methods

We utilized satellite remote sensing of fires, climatic models, and ASTER/ABoVE remote sensing resources in the following methods to assess the trends and contexts of short-interval fires.

### Reburning proportions

Fire occurrence for each year was taken from the Monitoring Trends in Burn Severity (MTBS) dataset, from 1984 to 2016 (https://www.mtbs.gov/). The MTBS dataset is the most comprehensive dataset for large fires, using a consistent methodology at a moderate resolution (30 m). We chose MTBS over the Alaska fire history database (AFHD) despite the shorter temporal coverage for two main reasons. First, the AHFD reports only perimeters (and so unburned inclusions within a fire perimeter are not distinguished from burned^37^); this can result in an over-estimation of burned areas (20–33% unburned^29^) and thus unknown proportions of reburning within overlapping perimeters. Second, the older AHFD perimeters can be inexact, resulting in potential overestimations of fire overlap (personal observation). While there are known issues with MTBS severity metrics^38^, here we use MTBS to distinguish only between burned and unburned sites. All severities < 2 were classified as unburned, all others burned. This is somewhat conservative but avoids erroneous overlap. Sampling points were uniformly spread across the study area (n = 215,553, approximately 2 km spacing). Salvage logging after an initial fire was not considered, as it is extremely limited in scope, constrained to near roads, and primarily occurred on fires that burned prior to the study period^39^. At each point, burn year(s) were extracted, along with slope, aspect, elevation (30 m resolution^40^), bioclimatic variables (BioClim^41^) and ecoregion (EPA Level 3^42^). Percentage of reburning was calculated as the fraction of points with 2 + burns compared to total points with burns in the observational record within the time period of observation years.

### Trends in short-interval fires overall

We used a temporal moving-window approach to examine trends in total reburning without confounding trends with increases in fire frequency. Ten and twenty-year reburn intervals were chosen because they represent a time period where the seed bank would likely not be mature, and thus a strong potential for a vegetation regime shift. Starting from the beginning of the observational period (1984), we calculated the reburning proportion for the interval (e.g., 1984–1993) for each year up to latest date for that interval of investigation (2016). As reburning rates should increase simply as a result of increasing fire, we utilized a randomized null model based on observed rates of fire overall during that time period (similar methods as^27^ in the Western US, see also^26^ for similar spatial, non-temporal approach in the boreal). The observed percent area burned over the reburn interval of interest (e.g., 1984–1993 for a 10-year window) was randomly distributed across vector space, then the observed percent area burned in the following focal year (e.g., 1994) was similarly distributed. Overlap between these two sets of burn locations was counted as a reburn, and percentage of total burned area calculated. This was repeated 10,000 times to generate means and 25th/75th percentiles of anticipated reburning percentage in the absence of any spatial interaction (the null model distribution). Before estimating trends, the response values were tested for short-term autocorrelation via the Box-Pierce test (all *p*-values were ≥ 0.05). Trends were estimated using the Theil-Sen estimator, a method that uses medians for a more conservative approach to small datasets.

### Trends in coniferous short-interval fires

The satellite-derived ABoVE dominant landcover map (30 m resolution^43^) was utilized to classify beginning-of-study forest type for all burned and reburn plots across the study area. While it would be ideal to have successional trajectories in the recently burned areas known prior to the second fire, those data are not available at scale, so we assume for the purposes of discussion that after a single fire in a boreal coniferous forest, conifers are likely regenerating irrespective of burn severity^21^. EPA classifications were collapsed down into coniferous forest, broadleaf forest, shrub/herbaceous, barren/sparsely vegetated, and wetlands (which include bogs and fens). To establish if coniferous forests were increasingly experiencing short-interval fires relative to other cover types, the temporal relationship between the proportion of conifers reburning (relative to all other cover types) was estimated using a linear regression weighted by the number of reburn points in a given year for the same 10- and 20-year intervals used in the overall reburn trend analysis.

### Predictability and context of short-interval fires

Conditional random forest modeling^44^ was used to establish the topo-climatic context for short-interval fires relative to single fires and determine if those factors do represent a unique climate-topography space. Conditional variable importance is an improvement over traditional random forest variable importance scores when predictor variables are highly correlated (e.g., climatic data), as it allows for conditioning on highly correlated variables, avoiding confounding correlation with importance^45^. We focused on out-of-bag error and relative variable importance. Climatic variables were taken from BioClim; topography variables extracted from a 30 m ASTER DEM. Aspect was transformed to a 0–1 scale prior to analysis^46^.

To explore which variables were most important in establishing a good fit and infer what characteristics of climate and topography were most associated with short-interval fires, we followed^44^, focusing on variable importance (permutation based) in overall model success. In this method, each predictor variable to be considered is randomized (one at a time) and the resulting mean decrease in accuracy in the model, if any, is seen as the quantification of the importance of that predictor variable. All variable importance metrics in machine learning frameworks exist on a marginal to partial perspective, where marginal importance is related to the effect of a predictor without taking others into account, and partial importance is the additional value added, independently, on top of the other variables in the model. We focus on the partial perspective here by conditioning each variable on other, highly correlated variables (*p* < 0.05) prior to calculating importance; this “removes” shared variance explained and results in a more independent metric of importance for each. The results are interpreted as general trends in the importance of precipitation, temperature, variability/seasonality, and topographic variables.

Of all points, 20% were set aside for an independent test set (n = 564). The forest was grown on the remaining 80% (n = 2252). To quantify the stability of overall accuracy and importance metrics, we grew 20 independent forests with 1000 iterations each, recording the conditional mean decrease in accuracy for each variable. All analyses were conducted in R 4.0.4^47^, primarily using the sp^48^, raster^49^, party^50^, permimp^50^, and NSM3^51^ packages and their dependencies.

## Supplementary Information

Supplementary Information.

## Data Availability

Code and data are available from Zenodo (10.5281/zenodo.6353983).
