# Supplementary material for: Short-interval fires increasing in the Alaskan boreal forest as fire self-regulation decays across forest types
Source: Sci Rep. 2022 Mar 22;12:4901. doi: 10.1038/s41598-022-08912-8 (PMC8941092; doi:10.1038/s41598-022-08912-8)
Supplement: Supplementary file 1 — Supplementary Information. [file 41598_2022_8912_MOESM1_ESM.docx]

**Supplementary material**

for

**Short interval fires increasing in the Alaskan boreal forest as fire self-regulation decays across forest types**

Authors:

B Buma, K Hayes, S Weiss, M Lucash

| 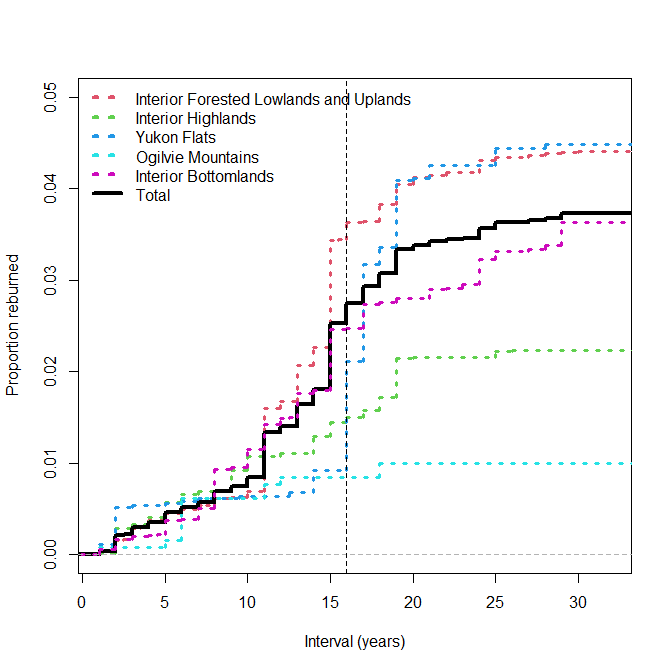 |
| --- |
| Figure S1. Empirical cumulative distribution function of the overall proportions of reburning compared to the interval between those fires, for the entire dataset. The vertical dashed line denotes the halfway point (16 years), where data becomes progressively more biased away from reburns (less potential opportunity). |

| 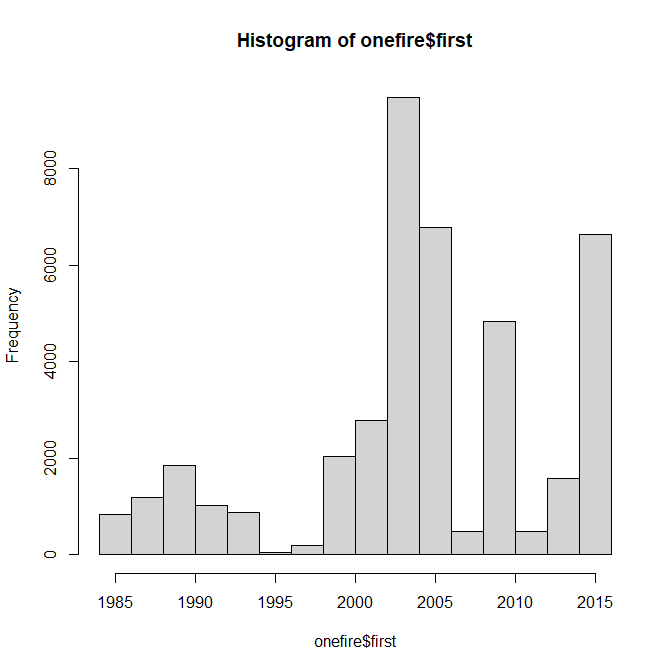 |
| --- |
| Figure S2. Histogram of locations with fires within the study area since 1984. |
